# Bacterial seed endophytes promote barley growth and inhibits *Fusarium graminearum in vitro*

**DOI:** 10.1186/s13104-024-06955-w

**Published:** 2024-10-03

**Authors:** Oyeyemi Ajayi, Suvir Grover, Belayneh Yimer, Marcus Vinje, Ramamurthy Mahalingam

**Affiliations:** 1 Cereal Crops Research Unit, USDA-ARS, 502 Walnut Street, Madison, WI 53726 USA; 2 Department of Genetics, University of Wisconsin-Madison, 425 Henry Mall, WI Madison, 53726 USA; 3 Small Grains and Potato Germplasm Research, USDA-ARS, 1691 S. 2700 W, Aberdeen, ID 83210 USA

**Keywords:** Barley, Seed endopyhtes, Bio-priming, Biocontrol, Fusarium

## Abstract

**Objectives:**

Seeds host microbes that function in plant growth and phytopathogen resistance. The aim of the work was to investigate total bacterial community in malting barley seeds and whether their bacterial seed endophytes have dual functional roles in plant growth-promotion and inhibition of *Fusarium graminearum*, the causative agent of Fusarium head blight (FHB) in barley. We used culture dependent and culture independent methods.

**Results:**

Phylogenetic classification of seed endophytic bacteria based on sequencing data identified *B. subtilis*, *B. licheniformis* and *B. pumilis* as predominant subgroups. Location driven divergence in bacterial endophytic communities was evident based on a clear separation of the samples from Crookston and other location samples. The bio-primed seeds using one hundred and seventy bacterial isolates showed that 3.5% (6/170) of the bacterial isolates conferred greater than 10% increase in both root length (RL) and shoot length (SL), while 19.4% (33/170) and 26.5% (45/170) showed RL and SL specific growth effects, respectively, relative to controls. Among the six bacterial isolates that increased RL and SL, five (#29, #63, #109, #124 and #126) also significantly inhibit the growth of *F. graminearum* based on in vitro assays. This study identified novel seed bacterial endophytes that could be further exploited for promoting growth during seedling establishment and as biocontrol for combating the devastating scab disease.

**Supplementary Information:**

The online version contains supplementary material available at 10.1186/s13104-024-06955-w.

## Introduction

Barley is an important cereal crop that is negatively affected by Fusarium head blight (FHB) disease resulting in about 80% yield loss worldwide [1, 2]. FHB is a destructive disease of barley caused by *Fusarium* species, particularly *F. graminearum*, and are known to produce mycotoxins that are harmful to human and animal health [3]. Efforts to boost yield and reduce disease pressure in crop plants have resulted in the increased use of synthetic fertilizers and other agrochemicals, with concomitant effects negatively impacting soil, air and water [4]. Seeds contain diverse microbial communities that can boost nutrient availability and uptake and enhance biotic and abiotic stress tolerance in crop plants [5]. Thus, seed microbes are beneficial novel alternatives for minimizing or replacing synthetic agrochemicals.

Earlier works reported that some beneficial microbes demonstrated both antifungal and plant growth promoting properties [6–9], and are attractive candidates for improving plant growth and boosting plant resistance to pathogen attack. Taking into consideration the above-mentioned benefits of bacterial seed endophytes, our goal was to evaluate malting barley seeds grown in different environments and identify bacterial endophytes with dual function roles in promoting seedling establishment and suppressing FHB disease.

## Main text

## Materials and methods

### Sample collection and surface sterilization of barley seeds

The malting barley genotypes and locations where they were grown are presented in Additional file 1: Table S1. One gram of barley seed samples was analyzed in quadruplicates to give a total of 80 samples (20 barley samples X 4 replicates = 80 samples total). For the barley seed surface sterilization, samples were first rinsed with sterile distilled water and washed with 70% ethanol for 3 min, followed by treatment with 1% sodium hypochlorite for 150 s as described previously [10, 11]. To evaluate the efficiency of the sterilization, 100 µl of the water from the last rinse from each sample was plated on nutrient agar and tryptic soy agar plates and incubated at 30^o^C for three days. Sterilized seeds were immediately processed and used for downstream analysis.

### Isolation of culturable seed endophytic bacteria

Isolation of bacterial seed endophytes from surface sterilized barley samples were conducted following methods described previously [11]. Briefly, one gram of sterilized seed samples was immersed in 10 ml of sterile water for 1 h and pulverized using sterilized mortar and pestle. Serial dilutions of the homogenized seeds were performed in sterile water to estimate the number of colonies. One hundred microliters (100 µl) of each serially diluted sample suspension was spread on nutrient agar (NA) and tryptic soy agar (TSA) plates and incubated at 30^o^C for 4–5 days, while the remaining ground samples were used for downstream DNA isolation. Morphological traits - colony size, color, form, elevation, margin, texture, and opacity of the purified bacterial isolates on NA and TSA were evaluated based on Berge’s Manual of Determinative Bacteriology [12]. The distinct isolates were transferred to NA plates for working stocks. Glycerol stocks (30%) were stored in -80^o^C for future use.

### DNA isolation and quantitative 16 S rRNA gene amplification

One gram of sterilized seed samples was pulverized using a sterilized mortar and pestle. DNA was isolated using DNeasy^®^ Plant Mini Kit (Qiagen, the Netherlands) according to the manufacturer’s protocol. DNA quality and purity were assessed using 0.8% agarose gel and Nanodrop spectrophotometer, respectively. Samples with A260/280 ratio of 1.7–1.8 were used for PCR amplification of the universal 16 S rRNA gene. Metagenomic DNA was diluted to a concentration of approximately 1 ng/µL, of which 2 µL were used as template following manufacturer’s recommendations as described in the Femto bacterial DNA quantification kit (Zymo Research, USA). All qPCR reactions were carried out in triplicates to estimate bacterial 16 S rRNA gene copy numbers. The qPCR was run on a QuantStudio 6 Flex (Applied Biosystems) and the 16 S rRNA gene copy numbers were estimated using genomic DNA from *E. coli* strain JM109 (Zymo Research, USA) as an internal standard.

### Molecular characterization of bacterial isolates

Genomic DNA was extracted from bacterial cells pelletized from liquid cultures using Zymo Research QuickDNA fungal/Bacterial Miniprep Kit (Irvine, California, USA), following the manufacturer’s protocol. The 16 S rRNA genes of the bacterial strains were amplified by PCR using the universal primer pair 27 F (5´ -AGAGTTTGATC-MTGGCTCAG- 3´) and 1492R (5´ -GGTTACCTTGTTAC-GACTT- 3´) [13]. The amplified PCR products were purified from the bands (approx;1500 bp) using Gel and PCR Clean-Up System (Promega, USA), and sequenced using long read amplicon sequencing. Identical bacterial sequences were retrieved using BLAST and were aligned in CLUSTALW. To determine the evolutionary relationships a phylogenetic tree was made using the Neighbor-Joining method (1000 bootstraps) in MEGA X program (version 10.1.7) [14].

### Microbiota analysis and statistical methods

Publicly available 16 S rRNA amplicon sequencing data under bioproject PRJNA1108745 were used for the seed microbiota analysis given that the same barley samples (Additional file 1: Table S1) were sequenced and submitted to the sequence read archive (only genotype Explorer from Casselton and Ithaca sequence reads were unavailable). Raw sequencing reads were denoised, joined, delineated into amplicon sequence variants (ASVs), and assigned taxonomy in the Qiime2 (v.2023.7) environment [15]. Principal Coordinate Analysis (PCoA) ordination plots including permutational multivariate analysis of variance (PERMANOVA, 999 permutations) test for Unweighted Unifrac was constructed using the phyloseq package [16] following methods described earlier [10].

### Seed biopriming for seedling development

Surface sterilized Conrad variety seeds were soaked in bacterial suspension (10^7^ CFU/mL) and sterile water (control) for 2 h. After soaking, the seeds were removed from the liquid, placed on sterile-wet 10” x 15” germination paper and gently rolled into tubes, placed in 1 L beakers half filled with sterile water and incubated in a growth chamber (Percival Scientific AR36L) for 7 days on a diurnal cycle with the following conditions: day (lights on) 5 am-11 pm, 22^o^C; night (light off) 18^o^C. One hundred and seventy isolates were tested and replicated twice with eight seeds per replicate for each bacterial isolate. Data on root and shoot lengths (RL and SL) were measured using imageJ. Clean area and frequent sterilization of gloves with 75% ethanol was maintained throughout the experiment to avoid contamination.

### In vitro antagonism against *F. graminearum*

Six bacterial isolates that performed well in enhancing RL and SL and one randomly selected isolate were tested for their antagonistic effect on a highly virulent *F. graminearum* strain, Fh1, in vitro, following methods described earlier [17]. Fh1 was isolated from barley, and its virulence confirmed on barley plants. Briefly, *F. graminearum* was grown on PDA plates at 25 °C for one week, and then 4.0 mm diameter agar with mycelia from the plate was placed in the center of another PDA plate. Single bacterial strains per plate were streaked approximately 3.0 cm equidistant from the center of the plate, and on one side of the fungus using a sterilized inoculation loop. The plates were then incubated at 28 °C for 5 days. The antagonistic effect of individual bacterial inoculum on *F. graminearum* was determined by measuring the diameter of the fungus on plates. In the control plates, sterile water was streaked in place of a bacterial isolate. Inhibition ratio and inhibition zone were measured as described previously [17] and statistically analyzed using the GLM procedure of SAS v.9.4.

## Results

**Fig. 1:**
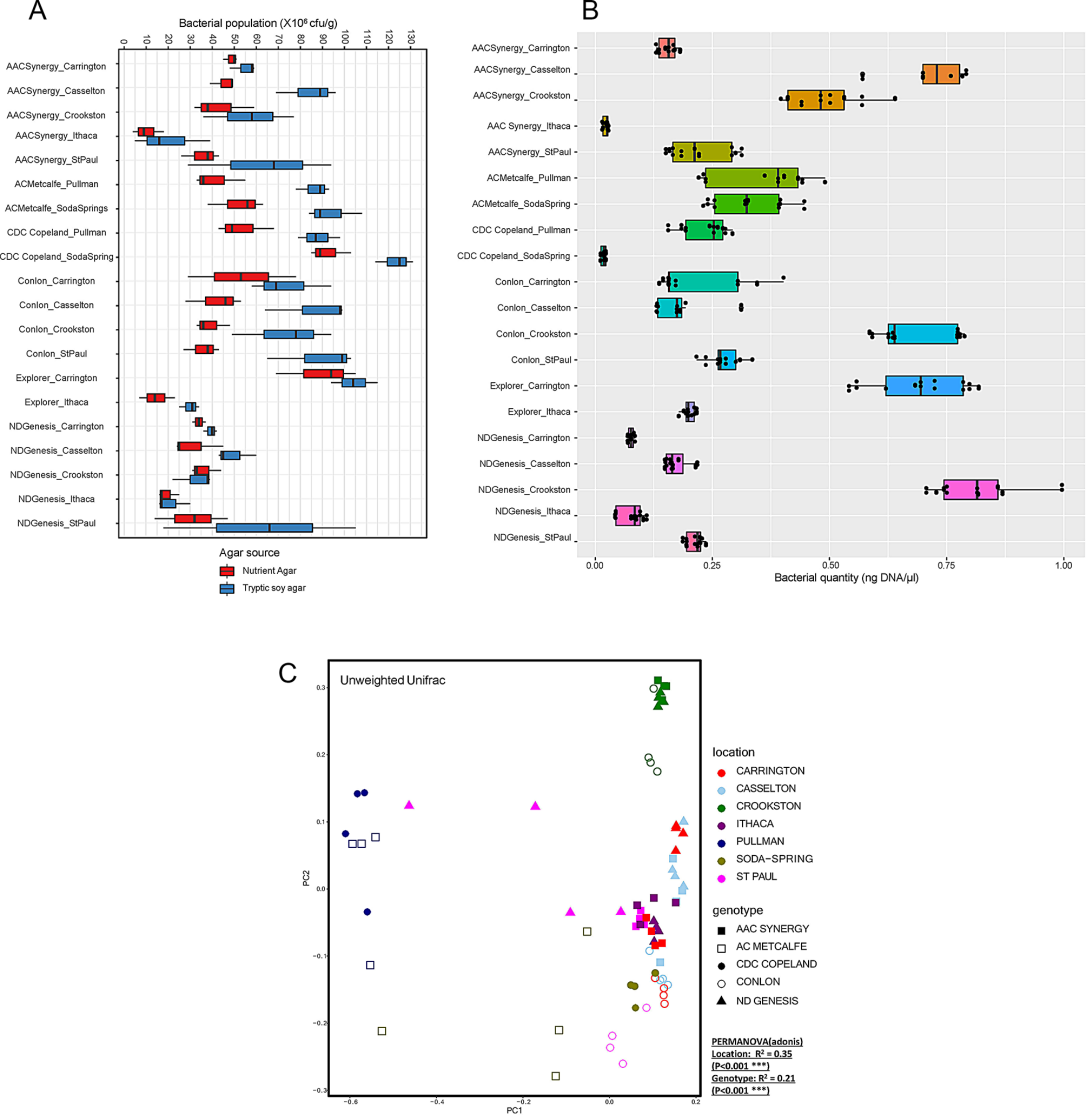
Comparative analysis of cultured and total bacterial community and diversity in the malting barley seed genotype-location samples. (**A**) Box plot representing the population of bacteria (cfu g^–1^) cultured in Nutrient Agar and Tryptic Soy Agar, (**B**) Total bacterial DNA based on 16 S rRNA gene quantification (ng DNA/µL), and (**C**) Beta diversity represented through principal coordinate analysis (PCoA) plots calculated using unweighted UniFrac of barley genotype-location samples

Total culturable bacterial community of malting barley seed endophytes showed higher number of culturable bacteria in TSA compared to NA. In addition, CDC Copeland from Soda Spring and Explorer from Carrington had the highest number of culturable bacteria while all barley genotypes originating from Ithaca had the lowest number of culturable bacteria with results for other genotype-location samples nestled in between the two extremes (Fig. 1A). For the bacterial isolates on NA plates, AAC Synergy and Conlon genotypes from Carrington, Casselton, Crookston, and St. Paul showed similar trend with the highest and lowest number of culturable bacteria found in Carrington and St. Paul, respectively (Fig. 1A). For the quantification of the total bacterial DNA (estimated using qPCR), ND Genesis from Crookston, MN location had the highest bacterial DNA while CDC Copeland from Soda Spring, ID had the lowest amount of bacterial DNA (Fig. 1B). Notably, all genotypes from Ithaca consistently showed lower amounts of bacterial DNA compared to the same genotypes from other locations (Fig. 1A **and** Fig. 1B).

Beta diversity measures based on Unweighted Unifrac distance revealed a significant grouping of samples with location and genotype significantly influencing the barley seed endophytic microbiome based on PERMANOVA estimates (Location, R^2^ = 0.35, *p* ≤ 0.001; genotype, R^2^ = 0.21, *p* ≤ 0.001) (Fig. 1C). CDC Copeland and AC Metcalfe from Pullman clustered separately from Soda-Spring while genotypes from Crookston location clustered separately from the remaining genotype-location samples. In addition, AAC Synergy and ND genesis appeared to cluster together for all locations, except Carrington and St. Paul (Fig. 1C).

**Fig. 2:**
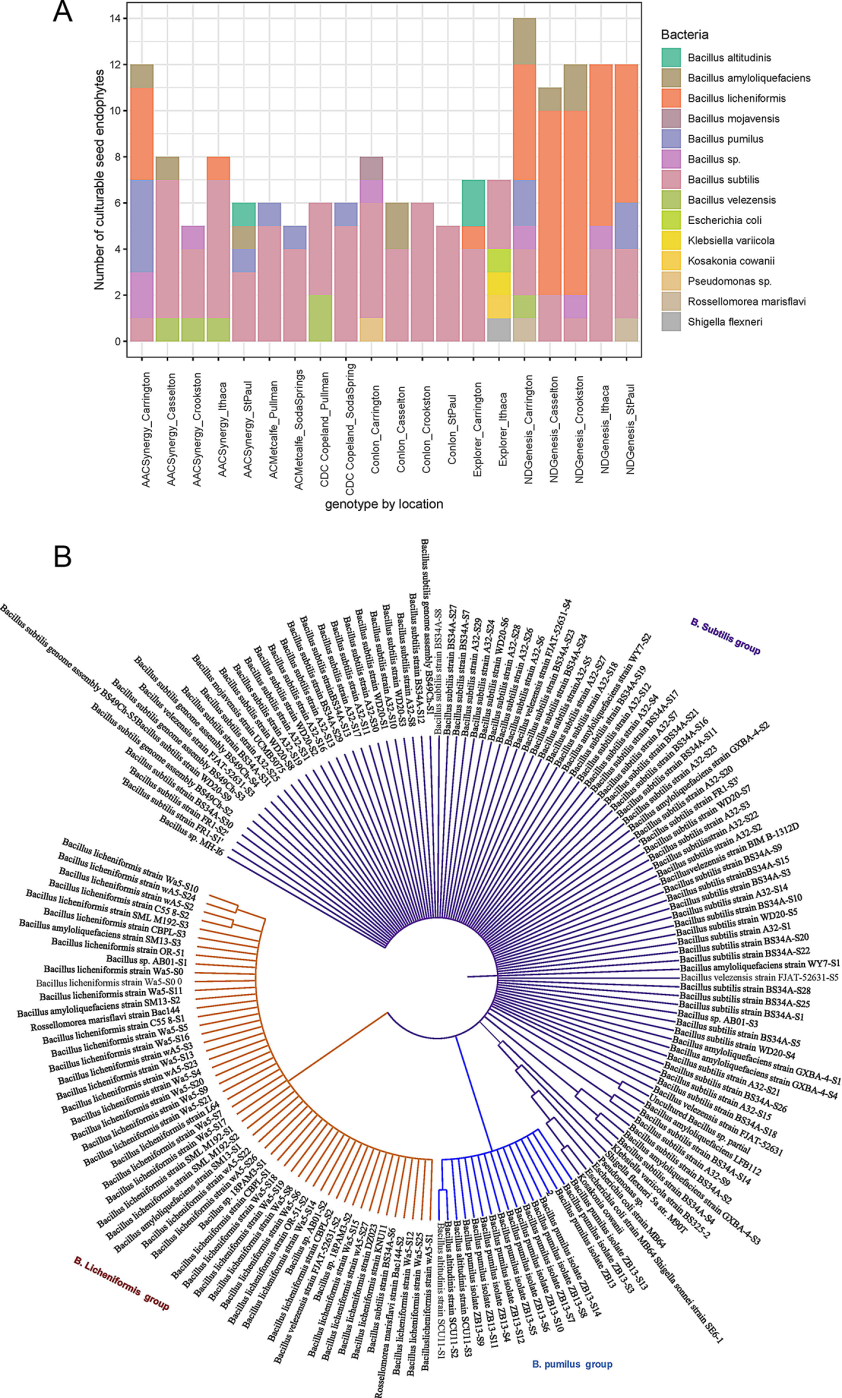
Cultured isolates and their phylogenetic analysis. (**A**) Number of culturable seed bacterial endophytes presented as stacked bar graph (**B**) Circular phylogenetic tree of one hundred and seventy (170) culturable bacterial isolates constructed using neighbor joining algorithm with 1, 000 bootstrap values

Seed bacterial endophytes were primarily enriched with genus Bacillus, with ND Genesis notably enriched in *B. licheniformis* (Fig. 2A). Based on phylogenetic classification, three main clusters were identified and enriched with *B. subtilis*, *B. licheniformis*, and *B. pumilis* subgroups (Fig. 2B), with other genera nestled in between these subgroupings.

**Fig. 3:**
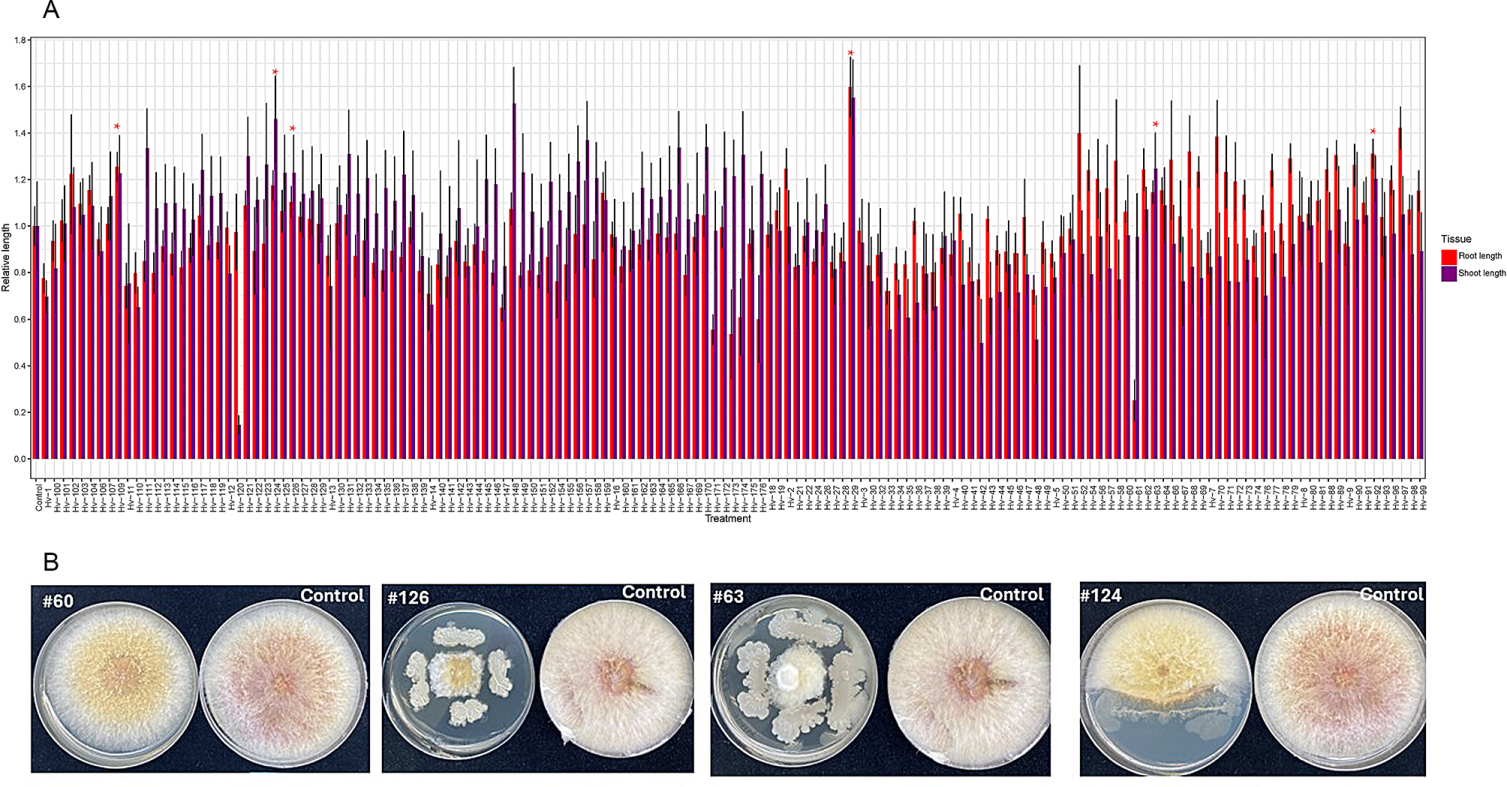
Seed biopriming for plant growth promoting assay and antagonistic effects of Fusarium head blight using culturable bacterial isolates. (**A**) Bar graph (mean ± s.d) of shoot and root length of one hundred and seventy cultured bacterial isolates relative to hydro-primed seeds (control) tested for plant growth promoting functions. Values for each biological replicate (sixteen biological replicates/treatment, see methods) were divided by the mean value of the control (mean normalized) and plotted. Red asterisks depict bacterial isolates with greater than 10% increase in both root and shoot length (**B**) Antifungal effects of bacterial isolates on the mycelium growth of *F. graminearum.* Note that #60, #63, #124 and #126 corresponded to Hv-60, Hv-63, Hv-124 and Hv-126 in Fig. 3A

For the seed priming experiment, varying effects of bacterial isolates on seedling growth 7 days post-inoculation was observed. Specifically, 3.5% (6/170) of the bacterial isolates induced greater than 10% increase in both RL and SL, while 19.4% (33/170) and 26.5% (45/170) were RL and SL specific in their growth effects, respectively, compared to the control (Fig. 3A**).** Further testing of the six isolates with greater than 10% increase in both RL and SL identified five bacterial isolates: #29, #63, #109, #124 and #126 that belonged to *B. subtilis* showed high antifungal effects on the mycelium growth of *F. graminearum in vitro* (Fig. 3B and Additional file 1: Table S2). The average inhibition ratio ranged from 65.5 to 70.5%, while the average inhibition zone was between 0.9 and 1.9 mm (Fig. 3B and Additional file 1: Table S2).

## Discussion

Plant growth promotion and disease management are vital aspects of sustainable agriculture. The understanding of the seed microbiome is key to improving plant health and fitness in diverse agroecosystems. Seed endophytes that successfully colonize seedlings are important research targets [18] due to their beneficial interactions with the host plant at all stages of its development. In this study, culture dependent and culture-independent methods revealed genotype and location specific effects on the seed microbiome composition, and these findings are consistent with prior studies [19–21]. In addition, we observed that location exerted a stronger effect on the seed endophytic microbiomes, with location explaining 35% of bacterial variance while host genotype explained 21% of the bacterial variance. An earlier work reported that the environment had stronger effects than the host plant genotype in shaping seed microbiomes [22]. This report supports our findings and could explain the observed lower bacterial abundances in all barley genotypes from Ithaca location compared to their counterpart from other locations. Clearly, these observations highlighted the need for breeders to be aware of the environment where the progeny seed originates from during seed propagation, as significant seed microbiome compositional shifts may result in varying seedling growth responses if planting seeds are sourced from different environments.

Seed bio-priming is an economically viable eco-friendly technique to stimulate the growth of beneficial microbes to induce disease resistance [23], and improve plant fitness from germination to maturity in crop plants such as wheat [24], maize [25], and rice [26]. In this study, we observed that seed bacterial endophytes impacted barley seedling growth in a tissue specific manner, with some isolates influencing either SL or RL. A few “high performing” bacterial isolates possessed dual functional roles, positively impacting both RL and SL, as well as suppressing the mycelium growth of *F. graminearum in vitro*. The importance of seed bio-priming in modern agriculture is well documented, as it involved bioagents that activate key defense mechanisms and enhance plant systemic resistance to phytopathogens [27]. Interestingly, all “high performing” bacterial isolates belong to the genus Bacillus, which is not surprising, given the reported multi-faceted beneficial roles of Bacillus in promoting plant growth [28], production of phytohormones [29] and antagonizing phytopathogens via antimicrobial production [30]. Future work tailored towards a systematic field study of these high performing bacterial isolates is warranted for the potential application in crop improvement and FHB disease management.

### Limitations

We acknowledge the need to test these bacterial isolates on seeds grown in the field to further evaluate their candidacy as potential biofertilizer or bio fungicides, an idea we are currently exploring.

## Electronic supplementary material

Below is the link to the electronic supplementary material.

Supplementary Material 1 **Table S1** Barley genotypes and where they are grown (location). The barley varieties used and geographical locations from where the seeds were collected for this study, **Table S2** In vitro antagonistic effect of bacterial endophytes on *F. graminearum*, the causative agent of Fusarium head blight in barley, Results of co-inoculation of promising bacterial endopyhtes and *Fusarium graminearum*. Antagonism was measured by size of inhibition zone and percentage of inhibition based on control.

## Data Availability

The 16S ribosomal RNA sequences of the 169 bacterial endophytes in this report are deposited in Genbank and their accession numbers are PP867134 - PP867303.

## Abbreviations

FHB: Fusarium head blight
RL: Root length
SL: Shoot length
PERMANOVA: Permutational multivariate analysis of variance
PCoA: Principal Coordinate Analysis
NA: Nutrient agar
TSA: Tryptic soy agar
