# Supplementary material for: Bacterial seed endophytes promote barley growth and inhibits Fusarium graminearum in vitro
Source: BMC Res Notes. 2024 Oct 3;17:289. doi: 10.1186/s13104-024-06955-w (PMC11451136; doi:10.1186/s13104-024-06955-w)
Supplement: Supplementary file 1 — Supplementary Material 1 Table S1 Barley genotypes and where they are grown (location). The barley varieties used and geographical locations from where the seeds were collected for this study, Table S2 In vitro antagonistic effect of bacterial endophytes on F. graminearum, the causative agent of Fusarium head blight in barley, Results of co-inoculation of promising bacterial endopyhtes and Fusarium graminearum. Antagonism was measured by size of inhibition zone and percentage of inhibition based on control. [file 13104_2024_6955_MOESM1_ESM.docx]

Table S1. Barley genotypes and where they are grown (location).

| Genotypes | Location | Sample name |
| --- | --- | --- |
| AAC Synergy | Carrington, ND | AAC Synergy_Carrington |
| AAC Synergy | Casselton, ND | AAC Synergy_Casselton |
| AAC Synergy | Crookston, MN | AAC Synergy_Crookston |
| AAC Synergy | Ithaca, NY | AAC Synergy_Ithaca |
| AAC Synergy | St Paul, MN | AAC Synergy_StPaul |
| AC Metcalfe | Pullman, WA | AC Metcalfe_Pullman |
| AC Metcalfe | Soda Spring, ID | AC Metcalfe_SodaSpring |
| CDC Copeland | Pullman, WA | CDC Copeland_Pullman |
| CDC Copeland | Soda Spring, ID | CDC Copeland_SodaSpring |
| Conlon | Carrington, ND | Conlon_Carrington |
| Conlon | Casselton, ND | Conlon_Casselton |
| Conlon | Crookston, MN | Conlon_Crookston |
| Conlon | St Paul, MN | Conlon_StPaul |
| Explorer | Carrington, ND | Explorer_Carrington |
| Explorer | Ithaca, NY | Explorer_Ithaca |
| ND Genesis | Carrington, ND | ND Genesis_Carrington |
| ND Genesis | Casselton, ND | ND Genesis_Casselton |
| ND Genesis | Crookston, MN | ND Genesis_Crookston |
| ND Genesis | Ithaca, NY | ND Genesis_Ithaca |
| ND Genesis | St Paul, MN | ND Genesis_StPaul |

Table S2. *In vitro* antagonistic effect of bacterial endophytes on *F. graminearum,* the causative agent of Fusarium head blight in barley.

| Genotype | Location | Bacterial isolate | Taxon | Growth inhibition | Inhibition ratio (%) | Inhibition zone (mm) | Culture medium |
| --- | --- | --- | --- | --- | --- | --- | --- |
| Conlon | Casselton, ND | #126 | *Bacillus subtilis* | Yes | 70.5a | 1.9a | TSA |
| Conlon | Casselton, ND | #124 | *Bacillus subtilis* | Yes | 70.0 ^a^ | 0.9 ^b^ | TSA |
| ND Genesis | St Paul, MN | #29 | *Bacillus subtilis* | Yes | 68.5 ^a^ | 0.9 ^b^ | NA |
| AAC Synergy | Ithaca, NY | #63 | *Bacillus subtilis* | Yes | 67.7 ^a^ | 1.9 ^a^ | NA |
| Explorer | Ithaca, NY | #109 | *Bacillus subtilis* | Yes | 65.5 ^a^ | 1.0 ^b^ | TSA |
| AAC Synergy | St Paul, MN | #92 | *Bacillus subtilis* | No | 29.0 ^b^ | 0.0 ^c^ | NA |
| AAC Synergy | Ithaca, NY | #60 | *Bacillus licheniformis* | No | 5.0 ^c^ | 0.0 ^c^ | NA |
|  |  | Control |  | No | 0.0c | 0.0 ^c^ |  |

Statistically significant differences (P≤0.05) in inhibition ratio and inhibition zone between tested isolates are indicated by different letter. NA = Nutrient agar; TSA= Tryptic soy agar. Inhibition ratio (%) = (C-E)/Cx100; where C = diameter of the control colony; E = the diameter of the treatment colony.
